# Larval dispersal in three coral reef decapod species: Influence of larval duration on the metapopulation structure

**DOI:** 10.1371/journal.pone.0193457

**Published:** 2018-03-20

**Authors:** Laura Sanvicente-Añorve, Jorge Zavala-Hidalgo, Eugenia Allende-Arandía, Margarita Hermoso-Salazar

**Affiliations:** 1 Instituto de Ciencias del Mar y Limnología, Universidad Nacional Autónoma de México, Mexico City, Mexico; 2 Centro de Ciencias de la Atmósfera, Universidad Nacional Autónoma de México, Mexico City, Mexico; 3 Cátedra CONACyT, Laboratorio de Ingeniería y Procesos Costeros, Instituto de Ingeniería, Universidad Nacional Autónoma de México, Sisal, Yucatán, Mexico; 4 Comisión Nacional para el Conocimiento y Uso de la Biodiversidad, Mexico City, Mexico; University of Sydney, AUSTRALIA

## Abstract

Most coral-associated decapod species have non-migratory adult populations and depend on their planktonic larvae for dispersal. This study examined the metapopulation structure of three decapod species with different pelagic larval duration (PLD) from twelve coral reef complexes of the Gulf of Mexico. The dispersion of larvae was analyzed through the use of a realistic numerical simulation of the Gulf of Mexico with the Hybrid Coordinate Ocean Model. To study the transport and dispersion of particles in near-surface waters, a particle-tracking subroutine was run using as input the currents from the model. The simulation consisted of the launch of 100 passive particles (virtual larvae) every 24 hours from each reef throughout five years, and tracked for as long as 210 days. Results indicated that species with a short PLD, *Mithraculus sculptus* (PLD 8‒13 days), had a weak connection among the reefs, but higher self-recruitment, especially on the narrow western shelf. The species with a longer PLD, *Dromia erythropus* (28‒30 days), had a stronger connection among neighboring reefs (< 300 km). Finally, the species with an even longer PLD, *Stenopus hispidus* (123‒210 days), had a wider potential distribution than the other species. Circulation on synoptic, seasonal and interannual scales had differential effects on the larval dispersal of each species. The metapopulation structure of *M*. *sculptus* and *D*. *erythropus* seemed to combine features of the non-equilibrium and the patchy models, whereas that of *S*. *hispidus* presumably fit to a patchy model. These findings support previous observations that indicate that species with longer PLD tend to occupy larger areas than species with short PLD, although recruitment of juveniles to the adult populations will also depend on other factors, such as the availability of suitable habitats and the ability to colonize them.

## Introduction

The term metapopulation was coined by Levins [1] and, in a brief way, it referred to a population of populations. In the ecological literature, this concept has been used to describe the dynamics of a species living in habitat patches occupied by discrete local populations [2, 3]. While local population dynamics depends on births and deaths, global metapopulation dynamics is driven by migration [4]. The first metapopulation model, proposed by Levins [1], described the dynamics of a species in a set of patches that could be occupied by colonization processes or could become empty through extinction events. This model assumed that habitat patches were all the same size and were equally isolated [5]. However, because empirical evidence suggests that metapopulation structure falls within a range of patch sizes and dispersal levels, ecologists tried to capture the variability in the spatial structure and connectedness of metapopulations and proposed four basic types of structure models [5–7]: *i)* classical Levins model, with patches of equal areas and low migration rates, *ii)* mainland-island, with a large patch and several small patches, in which the large patch is the major provider of colonists, *iii)* patchy, composed of small to large patches, in which high rates of dispersal effectively join the patches, and *iv)* non-equilibrium, composed of small, highly isolated patches. In nature, many populations combine features of more than one models [7].

Marine ecologists define metapopulations as a set of local distant populations linked through dispersal processes [8]. In benthic organisms, the planktonic larval phase is the main ‒and sometimes the only‒ way by which geographically separated populations connect [9]. Thus, the defining characteristics of metapopulations are *i)* that juveniles and adults of a local population do not encounter others from another population, and *ii)* that there is interchange among populations through larval dispersal [8]. Knowledge of the patterns of marine larval dispersal is key for the comprehension of a range of ecological, biogeographical, genetic and evolutionary processes, as well as for the design of marine protected areas [10–13]. The degree to which local populations exchange individuals, or connectivity, is crucial to an understanding of marine ecological processes [14]. Several studies suggest that metapopulation connectivity plays a critical role in the dynamics, phenotypic variability and genetic diversity of local populations [8, 13, 15, 16]. Furthermore, connectivity confers on the metapopulation network increased robustness and resilience in the face of human or natural perturbations [15, 17].

In marine ecosystems, decapods form a large diverse crustacean taxon and are key components of food webs [18, 19]. They have potentially wide larval dispersal and specific habitat choices [8]. Some few decapod species show direct development (no larval stages), while others display indirect development with pelagic larval phases differing in duration. For instance, in the bigclaw snapping shrimp *Alpheus heterochaelis* the larval duration is only four days, whereas in the spiny lobster *Jasus edwardsii* it is 12 to 24 months [20, 21].

During the larval stage, many benthic species display two phases: the precompetent period, in which the larvae are developing to a stage capable of settling, and the competent period, in which the larvae have developed sufficiently to be able to settle [22]. The overall duration of the larval stage affects overall dispersal potential [8, 23]. Coupling pelagic larval duration (PLD) of marine species with hydrodynamic models of passive particles may predict travel time, dispersal distance and pathways of larvae. For example, the dispersal potential of the pink shrimp *Pandalus borealis* in the Barents Sea, of only one-month larval phase, is at most 330 km from the source location [24]. For the spiny lobster *Panulirus argus*, whose larval duration is about six months, the furthest dispersal is about 1500 km in the Caribbean Sea, but if ontogenetic vertical migration is considered most larvae recruit within 400 km of their spawning site [25]; this suggests that larval behavior ‒associated with the prevalent oceanographic conditions‒ can also influence the dispersal distance of benthic species [14, 25, 26]. Unfortunately, vertical distribution and swimming abilities of decapod larvae in the water column remain unknown for most species.

Larval duration is also fundamental to an understanding of the geographical distribution of species [27]. Species with short PLD might be expected to have narrower geographic ranges than species with longer PLD, but the evidence is conflicting [27–29]. The relative influence of hydrological, ecological and evolutionary processes on the geographical range of species varies according to circumstances that are not always well understood [27, 30, 31]. This highlights the need for further empirical data, especially for less-studied groups such as crustaceans and other marine invertebrates.

Recently, interest has grown in the study of marine population connectivity. In the Gulf of Mexico, studies concerning connectivity have addressed limited areas regarding either a numerical or a genetic approach [32–35]. In this gulf, the patchy distribution of coral reefs provides ideal conditions for the study of metapopulation dynamics. Coral reefs offer suitable habitat structure for the existence of a number of reef-associated species. Among them, decapods have developed close ecological relationships with a variety of coral reef species. For instance, cleaner shrimps remove parasites and injured tissue from fish of several species, thereby contributing to the health of the coral fish community [36, 37]. Because of their low mobility, cleaner shrimps may congregate in a restricted area of the reef and form a 'cleaning station', a place frequently visited by strongly parasitized or injured fishes [36, 38]. Decapods may also use other living organisms as food and shelter. Sponge crab species carry upon their carapace sponge pieces or other organic materials to camouflage themselves from predators and to obtain their food by cutting some sponge fragments [39]. The emerald crabs seek refuge and protection among the tentacles of sea anemones [40]. Besides their ecological role in the ecosystems, these decapod species are commercially valuable ornamental aquarium resources [41]. In this study, we analyzed the population connectivity of three decapod species of different PLDs among the coral reef systems of the Gulf of Mexico: the emerald crab *Mithraculus sculptus* (PLD 8‒13 days), the sponge crab *Dromia erythropus* (28‒30 days) and the cleaner shrimp *Stenopus hispidus* (123‒210 days). We analyzed the role of hydrodynamic features in the dispersal pathways and metapopulation structure of species, and we investigated the relationship of the geographical distribution of species with their PLD.

## Material and methods

### Study area

The Gulf of Mexico is a Mediterranean-type basin bounded by the USA, Mexico and the island of Cuba [42]. It is connected with the Caribbean Sea by the Yucatan Channel and with the Atlantic by the Florida Straits [43]. The circulation in the Gulf of Mexico is affected by the Caribbean Current System: water enters through the Yucatan Channel, bringing most of the renewal water to the Gulf, and flows out through the Florida Straits [43, 44]. On the eastern side, water flowing into the gulf forms the Loop Current, a clockwise flow that exhibits a variable northward penetration into the gulf, even into the outer West Florida Shelf [45–47]. The Loop Current sheds anti-cyclonic (clockwise rotating) eddies, which travel westward and eventually dissipate near the continental shelf [48]. Thus, circulation patterns in the entire Gulf of Mexico are strongly affected by the Loop Current and its associated eddies [49]. Continental water discharges and wind stress also affect the water transport, especially over the shelves [49–51].

**Fig 1:**
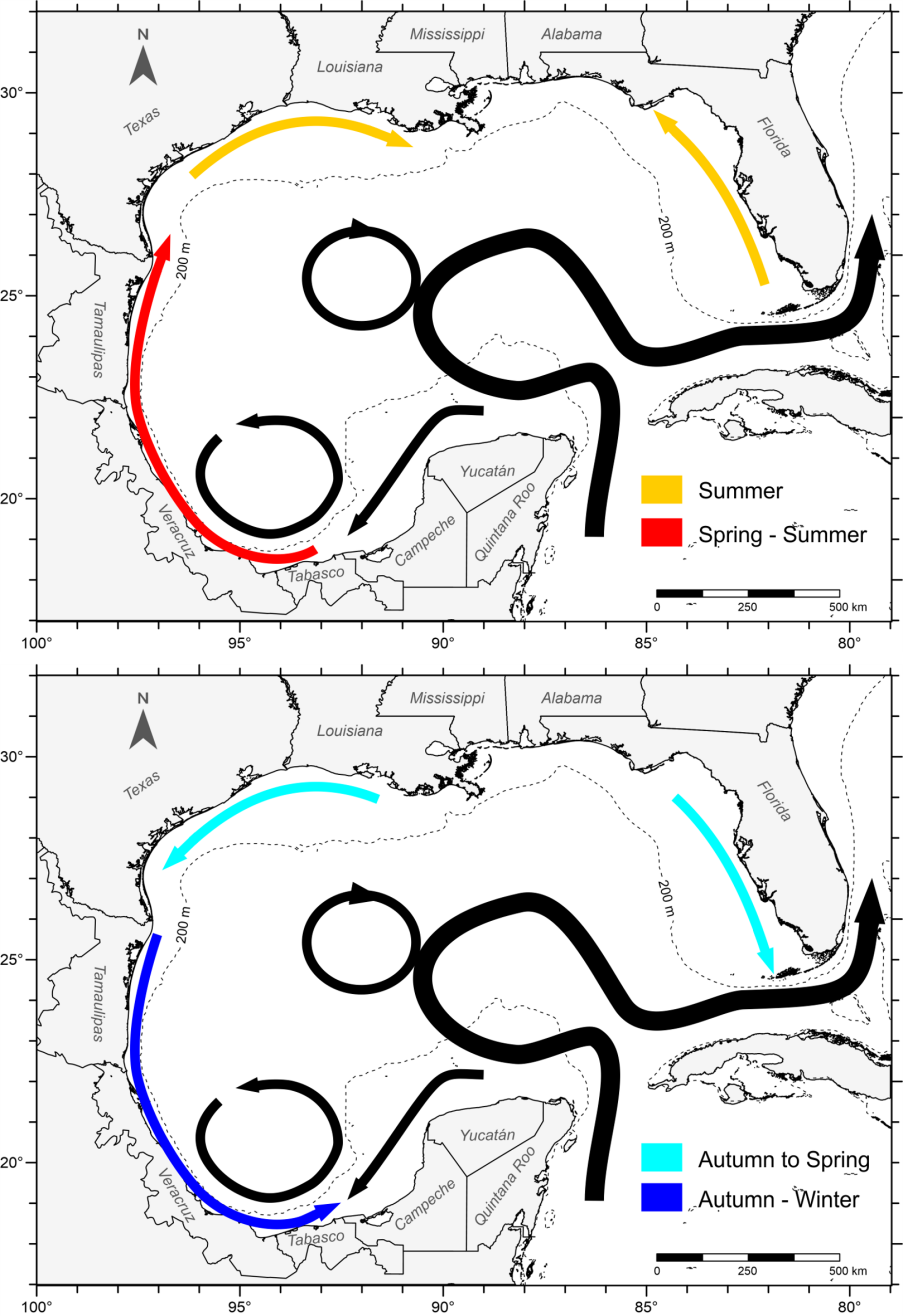
Main surface circulation patterns in the Gulf of Mexico.

In general, shelves bordering the Gulf of Mexico experience strong continental water discharges. The orientation of the dominant wind in relation to the main axes of the shelves is highly variable [52], and this causes seasonal differences in the circulation patterns. Circulation over the western shelf is mainly anticyclonic during the spring-summer, and cyclonic in autumn-winter [53]. Over the inner northern Texas and Louisiana shelves, it is mainly eastward during summer, and westward and southwestward the rest of the year [49, 53]. On the West Florida shelf there is a northwestward circulation during summer and southeastward the rest of the year [51]. Over the Campeche Bank the circulation is anticyclonic throughout the year [53]. This pattern causes two opposite currents to meet in the southernmost gulf and results in an offshore current during this period [53] (Fig 1).

**Fig 2:**
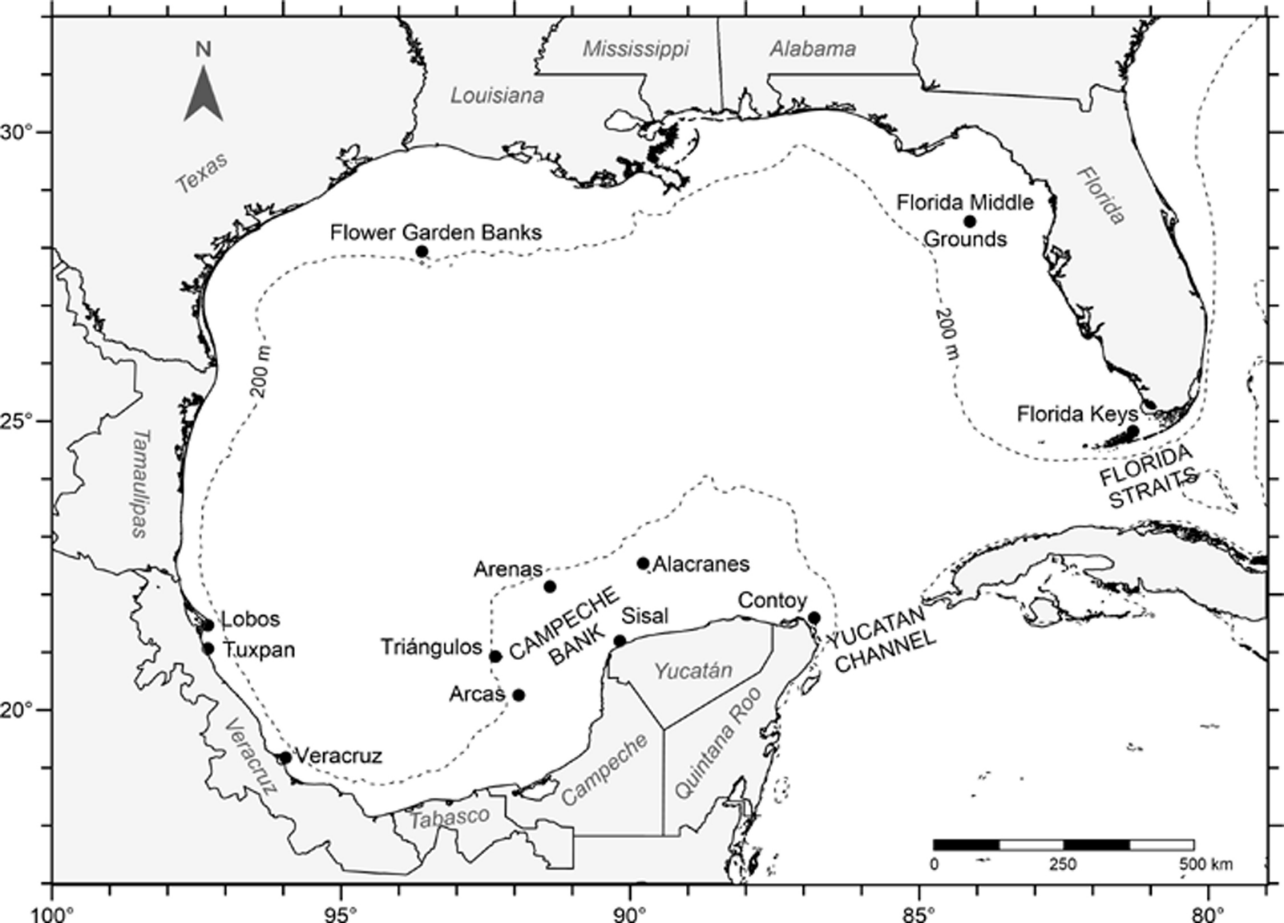
Geographical location of the twelve coral reef systems within the Gulf of Mexico.

Coral reefs in the Gulf of Mexico are patchily distributed and differ in size and in distance from the shore. Of the 264,009 ha of coral surface area, 63% lies over the Florida shelf and only 15% in the southern region [54]. The twelve reefs studied here are, in an anticlockwise direction and beginning from the north-easternmost reef: Florida Keys, Florida Middle Grounds, Flower Garden Banks, Lobos, Tuxpan, Veracruz, Arcas, Triángulos, Arenas, Sisal, Alacranes and Contoy (Fig 2). The first three systems are under the jurisdictions of the USA, and the other nine are Mexican.

### The hydrodynamic model

The ocean circulation in the Gulf of Mexico was simulated with the Hybrid Coordinate Ocean Model (GOM-HYCOM). This is a primitive equation ocean model that combines the sigma coordinates in shallow coastal waters, isopycnic vertical coordinates in the open stratified ocean, and *z*-level coordinates in the weakly stratified upper-ocean mixed layer [55]. The reanalysis GOM10.04 –exp_20.1 was used in this study. It was run with 20 vertical layers, a horizontal resolution of 1/25 degree and boundary conditions from the 1/12 degree Atlantic HYCOM (http://hycom.org). It uses the Navy Coupled Ocean Data Assimilation (NCODA) system for assimilation with model forecast as first guess [56]. NCODA assimilates *in situ* and satellite sea surface temperature, sea surface height from satellite altimeter, *in situ* vertical temperature and salinity profiles from XBT, as well as ARGO floats.

Daily outputs of a five-year period (2006 to 2010) were used to simulate the current circulation of surface waters. Larval dispersal was simulated off-line with a 2-D Lagrangian particle-tracking formulation coupled to the GOM-HYCOM circulation model. Trajectories of particles (virtual larvae) were estimated by integrating the following system [57].

$$\frac{dx}{dt}=u_{*}+u_{d}$$

$$\frac{dy}{dt}=v_{*}+v_{d}$$

The method used to solve the integral was a second-order Runge Kutta method with a time step of 6 hours. In this system, (*x*, *y*) is the particle position, (u_*_, v_*_) is the fluid velocity at the particle position estimated by a bilinear interpolation from the model velocity field grids, and (*u*_*d*_, *v*_*d*_) represents random velocities normally distributed (mean equals zero; variance, one) multiplied by a coefficient in order to incorporate diffusion in the circulation model. The diffusion is computed as *u*_*d*_ *= R*_*1*_*A* and *v*_*d*_ *= R*_*2*_*A*, following Döös and Engqvist [58] and Döös et al. [59]. Here, *R*_*i*_ (*i* = 1, 2) is a random number with a normal distribution and $A=\frac{b}{∆t^{\frac{1}{3}}}$ modulates the diffusion scale. In the simulations performed in this study, *b* = 1.3925, Δ*t* = 21600 s, and *A* = 0.05. With this formulation, the diffusion does not depend on the choice of Δ*t*. Validation of the HYCOM model regarding the three-dimensional ocean temperature, salinity, and currents, as well as the surface mixed layer and location of mesoscale features can be found in Metzger et al. [60].

The larval dispersal simulation consisted in releasing 100 passive particles every day from the center of each reef into surface waters for five years, and each virtual larva was tracked for as long as 210 days. Hence, 182,600 particles were released per reef during the five-year period. A sensitivity analysis testing different numbers of particles for the calculation of connectivity values revealed that as the number of particles increased, connectivity values tended to stabilize. Hence, even the release of 80 particles per day would be enough to give an estimation of connectivity values for the sites studied.

In order to prevent bias in the results, several conditions were imposed on the dispersal model. Thus, if a particle was outside the model domain, then the particle was returned to the previous position and moved again following the procedures described by Zelenke et al. [61].

The use of surface currents data for the larval dispersal simulation was prompted by the lack of empirical data concerning the ontogenetic vertical distribution of the target species, and by the fact that zooplankton biomass in the southern gulf is greater in the upper 30 m layer [62]. A correlation coefficient (> 0.93) between velocity data for the surface and for the 30 m depth revealed a highly consistent current direction in the upper 30 layer [33]. Also, *in situ* observations of two of us (LSA, MHS) in the southern reefs indicate the occurrence of gravid decapod females throughout the year, which is why particles in the present study were released all year.

### Connectivity matrices and target species

The degree of connectivity among reefs was expressed as the percentage of particles released from one site that arrived at another site, within a certain period [63]. In a connectivity matrix, the rows correspond to the starting sites of particles, and the columns to the endpoints. The diagonal cells of these square matrices represent an estimation of the self-recruitment of species, that is, the percentage of particles retained in the starting site. An event was considered successful if particles arrived within a circular area of 10 km radius centered on the recipient reef. A site was identified as a source if emigrants (percentage of particles successfully emitted by the site) exceeded immigrants (percentage of particles successfully received), or as a sink in the opposite case.

The target species differ in PLD and global distribution. *Mithraculus sculptus* (PLD 8‒13 days) is a small majid crab found from the Bahamas and southern Florida to northern Brazil, most abundant in back reef environments [64]. *Dromia erythropus* (28‒30 days) is distributed in the western Atlantic from Bermuda and Florida to Brazil and the Ascension and Saint Helena islands, including the Gulf of Mexico and the West Indies [65, 66]. *Stenopus hispidus* (123‒210 days) is a reef-associated cleaner shrimp with a worldwide distribution [67]. For each of these species, local occurrence of adult populations over the twelve reefs was documented from the Global Biodiversity Information Facility (GBIF) data base (http://data.gbif.org).

During simulations, particles (virtual larvae) remained in the water until the end of their PLD. The competent period of each species was considered as extending from the first to the last day of settlement ability, as reported in the literature [37, 68, 69]. Thus, we took six days for *M*. *sculptus*, three days for *D*. *erythropus* and 88 days for *S*. *hispidus*. Owing to the long competent period of *S*. *hispidus*, we also considered four different scenarios for this period: two of 44 days each (from the 123^th^ to 166^th^ day, and 167^th^ to 210^th^ day), and two of 10 days each (123^th^ to 132^th^ day, and 201^th^ to 210^th^ day). Successful events in different scenarios were independent among them; that is, the arrival of a particle in a period does not influence its arrival in another period.

## Results and discussion

### Larval dispersal and metapopulation structure of species

#### Mithraculus sculptus

**Fig 3:**
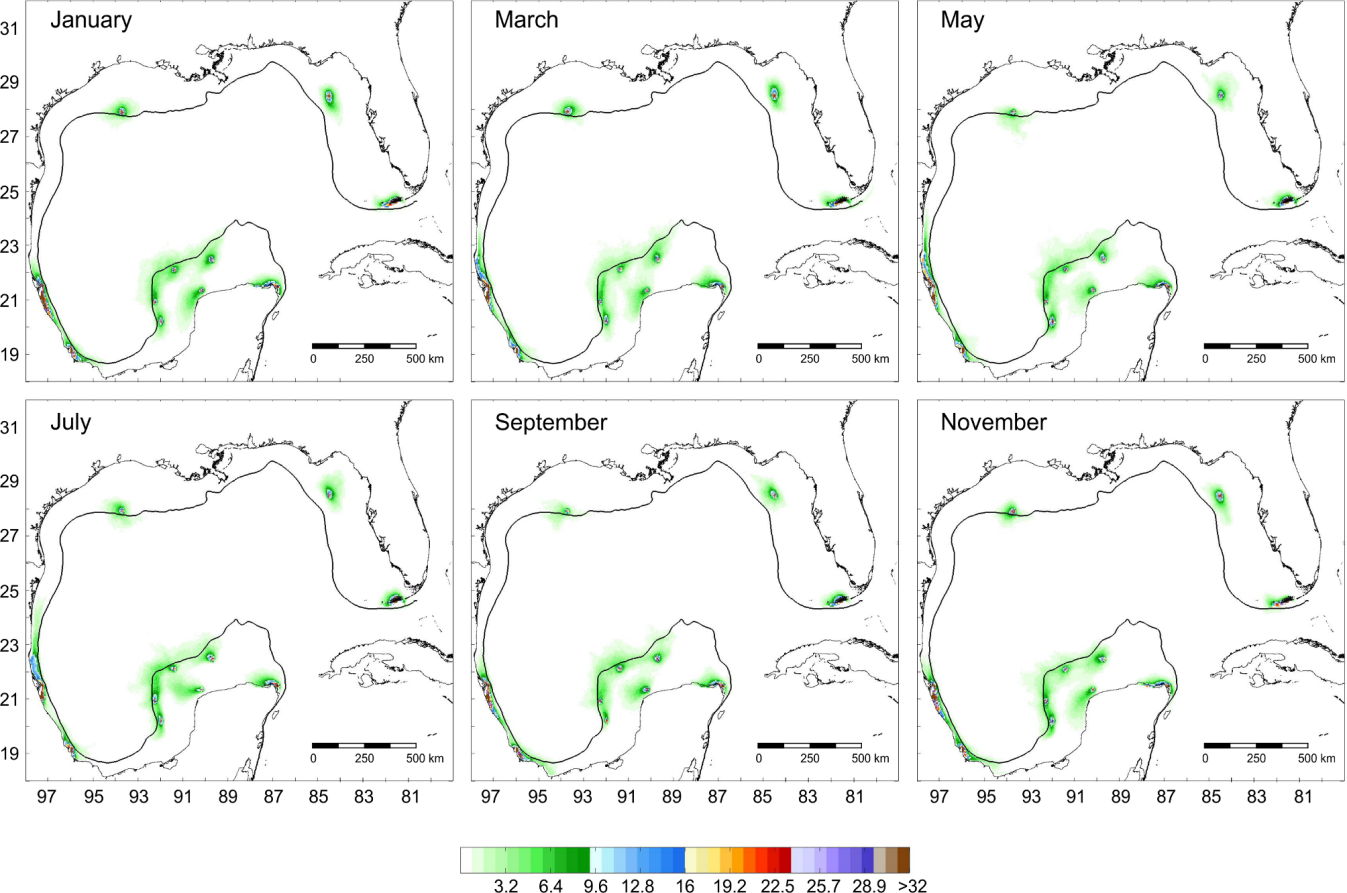
Potential dispersal of *Mithraculus sculptus* over twelve coral reef systems in the Gulf of Mexico. Values represent a percentage of particles related to the maximum number of particles per unit area.

**Fig 4:**
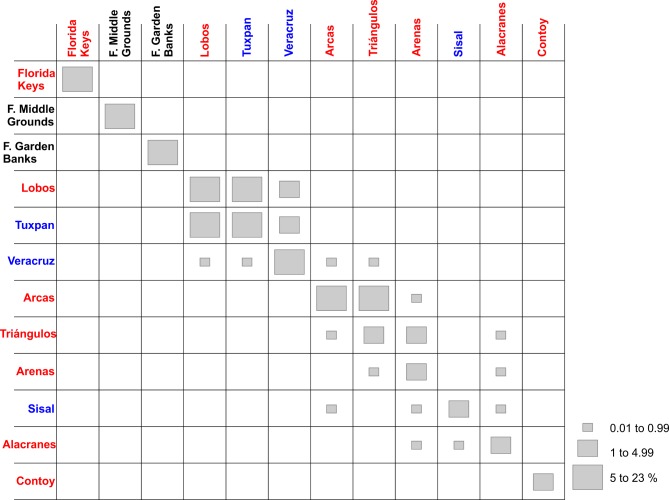
Connectivity matrix of *Mithraculus sculptus* among twelve coral reef systems in the Gulf of Mexico. Values represent the percentage of larvae arriving from the starting sites (rows) to the final destinations (columns). Red, recorded in adult stage in this reef; blue, potential colonization from the red sites; black, neither recorded as adult nor potentially colonized.

**Fig 5:**
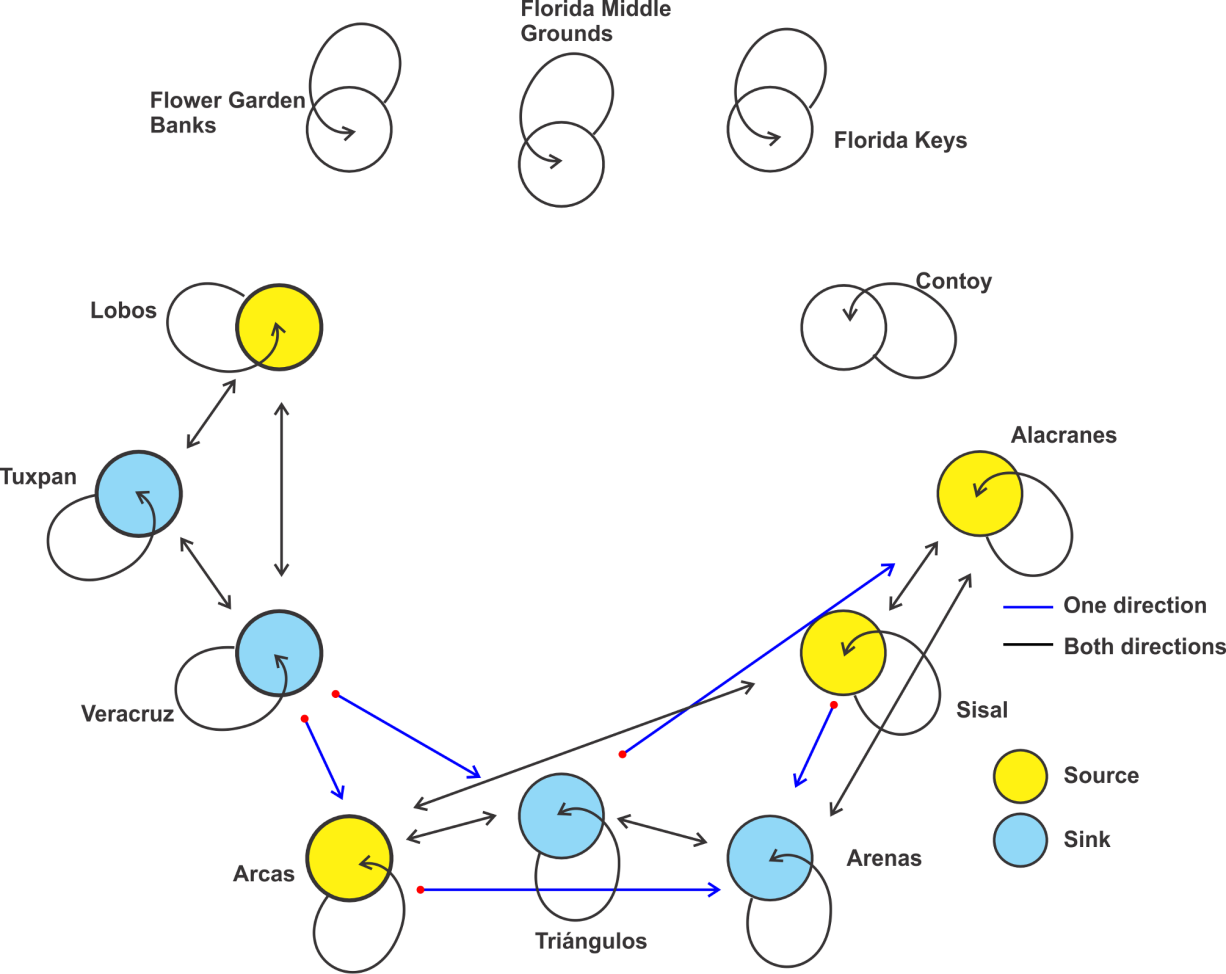
Schematic representation of the metapopulation structure of *Mithraculus sculptus* in the Gulf of Mexico.

As adult, this species has been recorded on six reefs in the gulf, but its dispersal suggests that the species is able to reach Tuxpan, Veracruz and Sisal (Figs 3 and 4). Simulations of the larval dispersal showed that its metapopulation structure seems to combine features of the patchy model in the southern region, and of the non-equilibrium model in reefs of the northern gulf and Contoy. A source-sink dynamics is expressed in the southern gulf: while Lobos, Arcas, Sisal and Alacranes function as sources; Tuxpan, Veracruz, Triángulos and Arenas function as sinks (Fig 5). According to Puckett and Eggleston [70], source and sink subpopulations contribute positively or negatively, respectively, to the persistence of the metapopulation; however, when local retention is higher than the contribution from immigrants, local demographic rates gain importance. This could be the case in this species.

In the southern gulf, reefs over the western shelf (Lobos, Tuxpan and Veracruz) connect only weakly with those of the Campeche Bank (Arcas, Triángulos, Arenas, Alacranes, Sisal) via Veracruz to Triángulos and Veracruz to Arcas (Figs 4 and 5). The minimum duration required for passive drifters, 9 days from Veracruz to Triángulos and 10 days from Veracruz to Arcas [33], explains the low connectivity. In the east–west direction (Arcas to Veracruz), there is no connectivity between those reefs because of the direction of the main currents and the short PLD. The subpopulations in the northern gulf have no connectivity with those in the southern part (Fig 5), because of the short pelagic larval phase. High self-connectivity values were observed over the western shelf. The main coastal circulation pattern on the western shelf is altered by episodes of an alternating current direction along the shelf, lasting from two to 15 days and driven by the passage of atmospheric systems [71]. These changes in the current direction could be the main cause of the high recruitment values of *M*. *sculptus*.

#### Dromia erythropus

**Fig 6:**
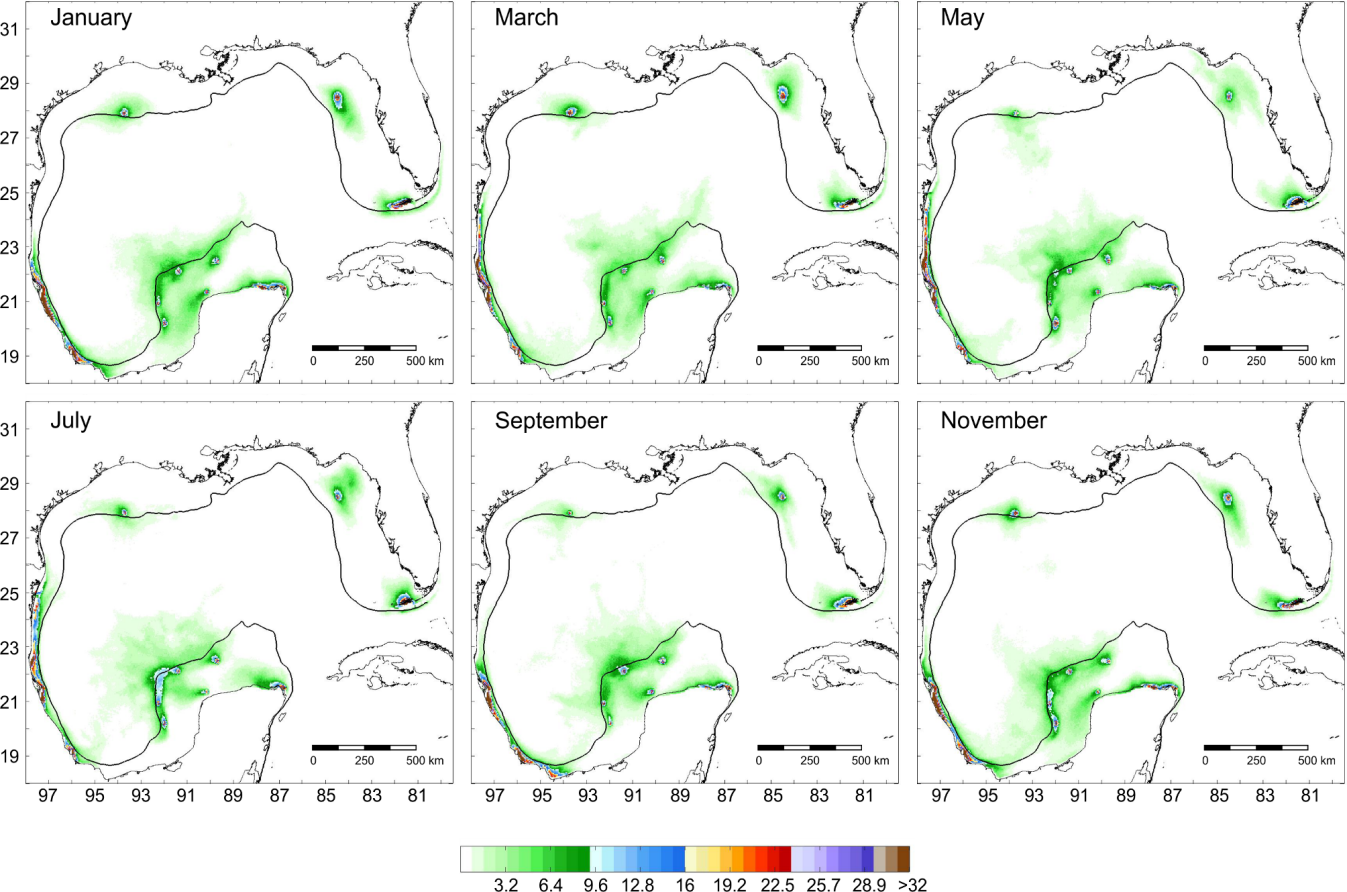
Potential dispersal of *Dromia erythropus* over twelve coral reef systems in the Gulf of Mexico. Values represent a percentage of particles related to the maximum number of particles per unit area.

**Fig 7:**
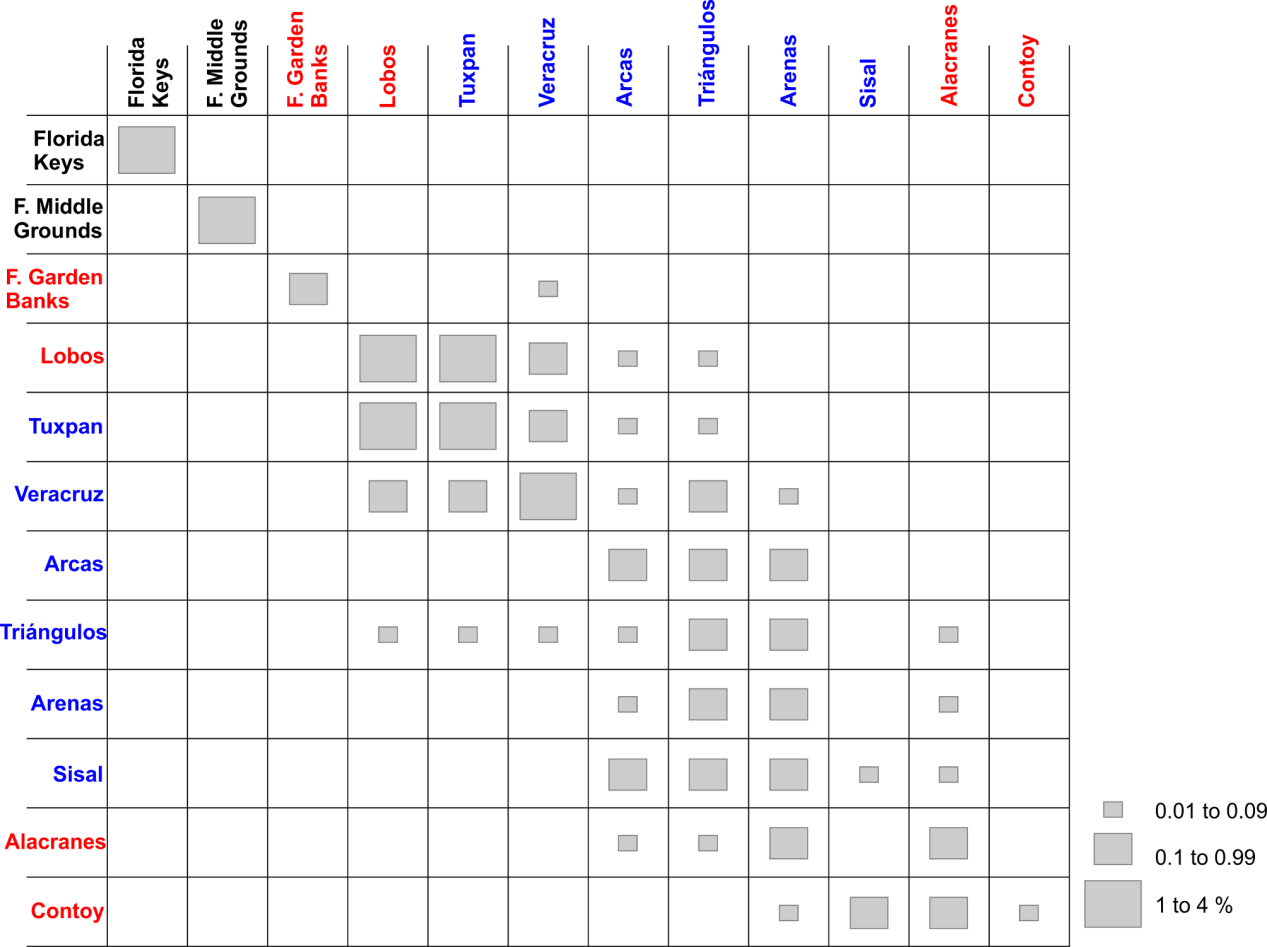
Connectivity matrix of *Dromia erythropus* among twelve coral reef systems in the Gulf of Mexico. Values represent the percentage of larvae arriving from the starting sites (rows) to the final destinations (columns). Red, recorded in adult stage in this reef; blue, potential colonization from the red sites; black, neither recorded as adult nor potentially colonized.

Adults of this species inhabit coral reefs and other hard substrates, at a depth of sea water up to 360 m [72]. GBIF database shows that adults of *D*. *erythropus* have been recorded on four reefs (Flower Garden Banks, Lobos, Alacranes and Contoy) of the Gulf of Mexico, but its dispersal potential suggests that the species can reach all the southern-region reefs (Figs 6 and 7). Numerical simulation indicated that Florida Keys and Florida Middle Grounds remain isolated from the other reefs. However, this does not mean that the species is completely absent from those reefs; perhaps it has not been registered in the database. To occur on those reefs, the source of larvae would be self-recruitment (Fig 7).

**Fig 8:**
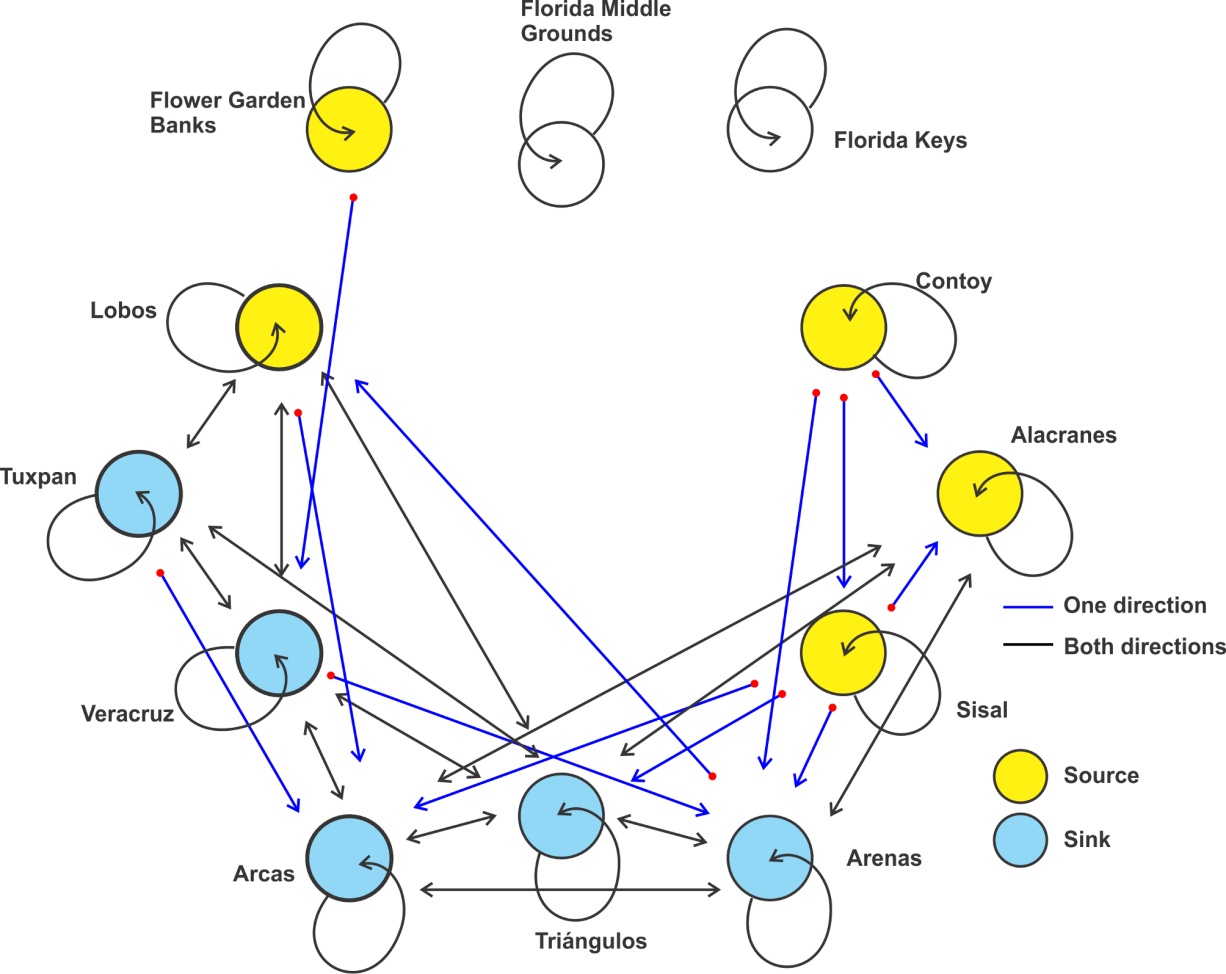
Schematic representation of the metapopulation structure of *Dromia erythropus* in the Gulf of Mexico.

Larval dispersal simulation suggests that the metapopulation structure of *D*. *erythropus* may combine the non-equilibrium model over the Florida shelf, with a patchy model in the remaining area (Fig 8). In the southern gulf, source-sink dynamics revealed a slightly higher number of migrants than of self-recruits, revealing the importance of connectivity in the persistence of the metapopulation [70]. In the southeastern side, Contoy, Alacranes and Sisal are important source sites for the reefs over the Campeche Bank, and Lobos fulfils the same function for the reefs over the narrow western shelf (Fig 8). The connection between the northern and southern gulf is via Flower Garden Banks to Veracruz. Connectivity is high among reefs over the western shelf (with an inter-reef-distance < 300 km); but they connect only weakly with the reefs over the Campeche Bank (Fig 7); this weakness (< 0.2%) may be due to the confluence of two directionally opposite currents that occurs during the fall‒winter period in the southernmost part of the Gulf of Mexico [53]. The numerical connectivity study of Johnson et al. [32] showed that only a small portion (1%) of the particles released over the Campeche Bank arrived in shelf waters elsewhere in the gulf after 31 days of drifting, indicating a limited connectivity between the southeastern and the southwestern gulf.

Results of the numerical simulation indicated high self-recruitment of *D*. *erythropus* over the narrow western shelf (Lobos, Tuxpan and Veracruz). This area has the highest self-recruitment values for a range of PLDs: 0‒15, 16‒25 and 26‒35 days [33]. In addition to the short-term synoptic atmospheric events described above, reversal in the mean seasonal current direction over the narrow western shelf [53] is probably an alternative cause of the self-recruitment. Metapopulation structure of this species is of high importance in the study of the connectivity of marine communities because its PLD represents a common dispersal period in marine species [73].

#### Stenopus hispidus

**Fig 9:**
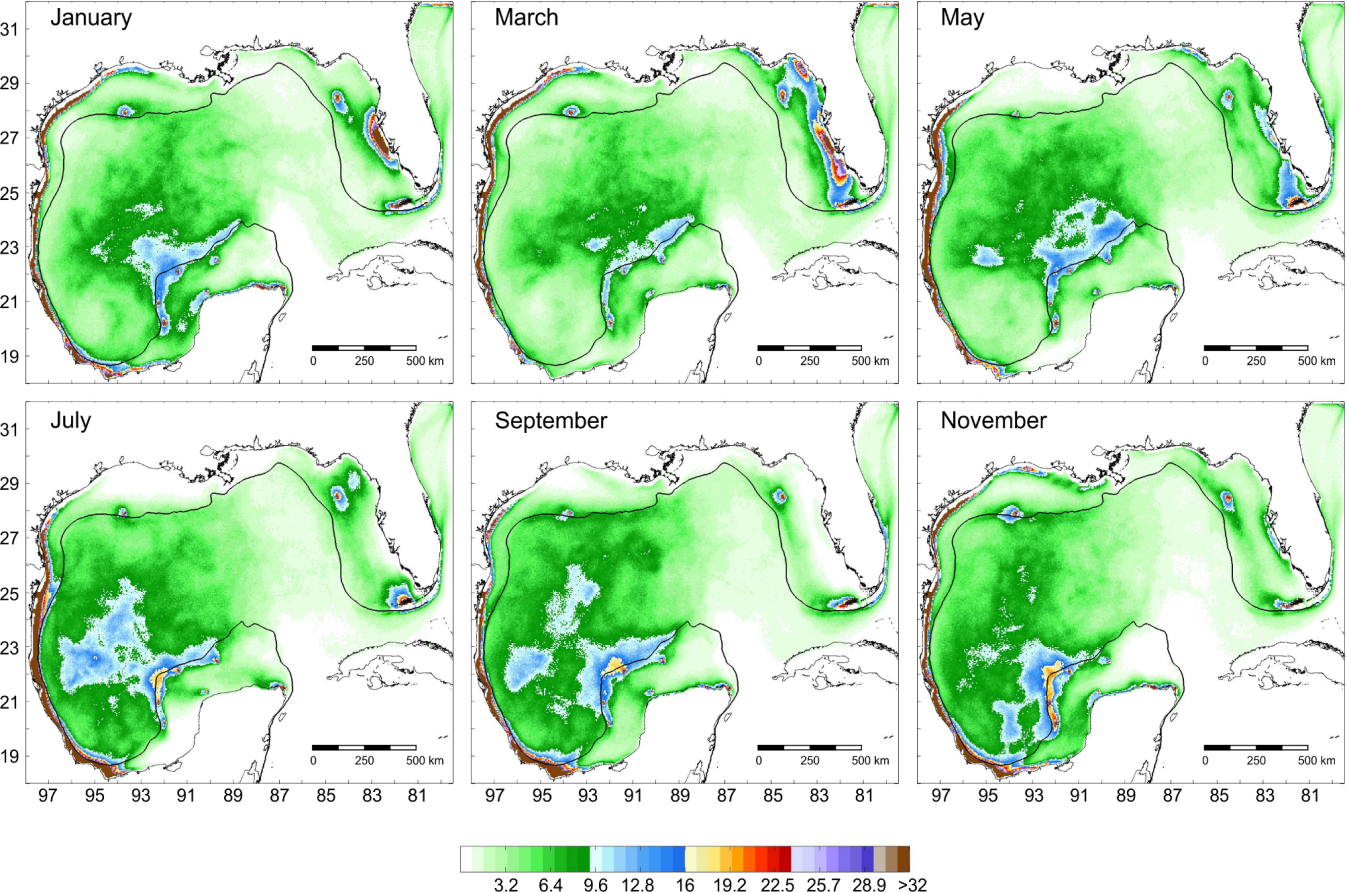
Potential dispersal of *Stenopus hispidus* over twelve coral reef systems in the Gulf of Mexico. Values represent a percentage of particles related to the maximum number of particles per unit area.

**Fig 10:**
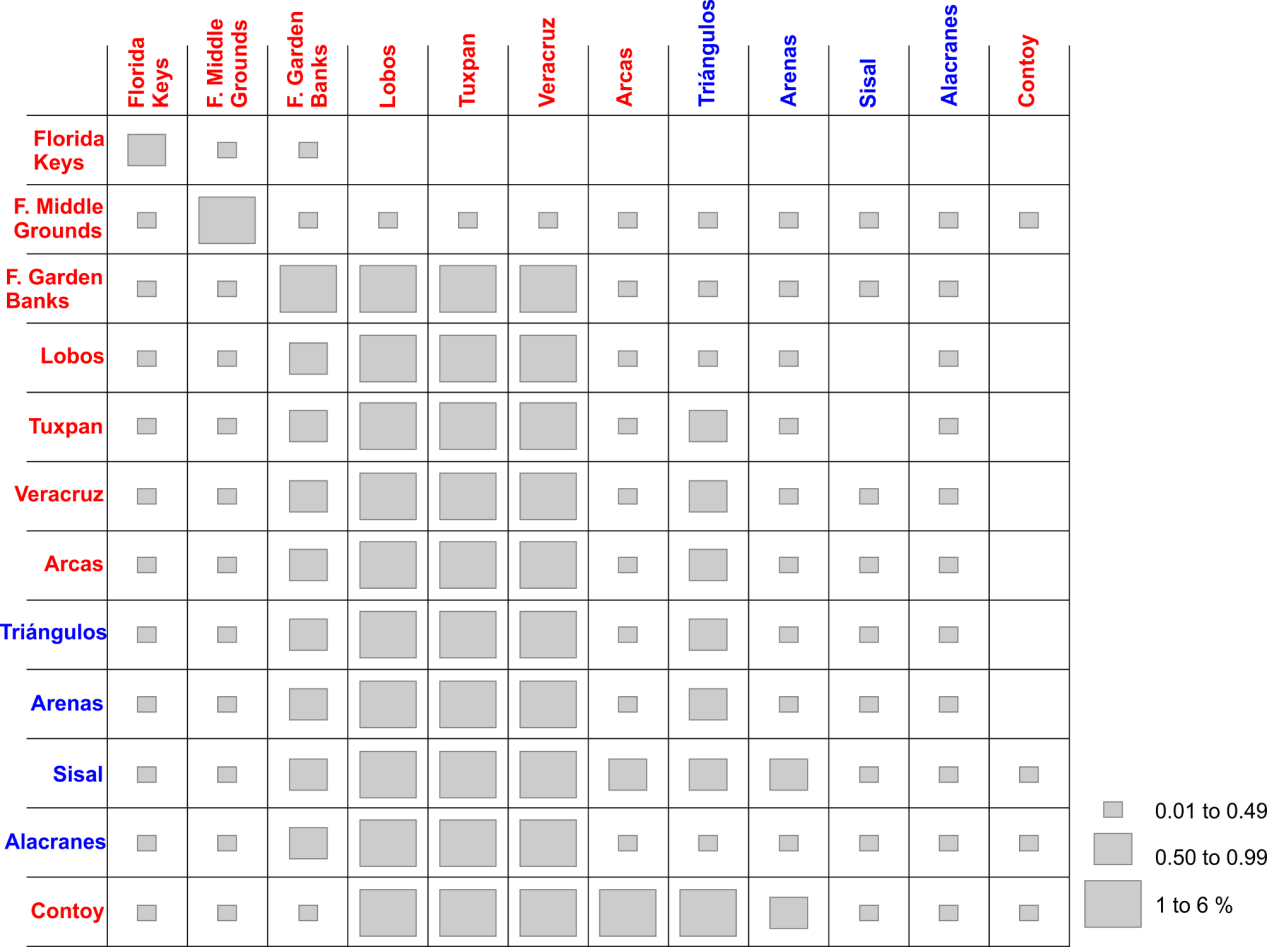
Connectivity matrix of *Stenopus hispidus* among twelve coral reef systems in the Gulf of Mexico. Values represent the percentage of larvae arriving from the starting sites (rows) to the final destinations (columns). Red, recorded in adult stage in this reef; blue, potential colonization from the red sites.

**Fig 11:**
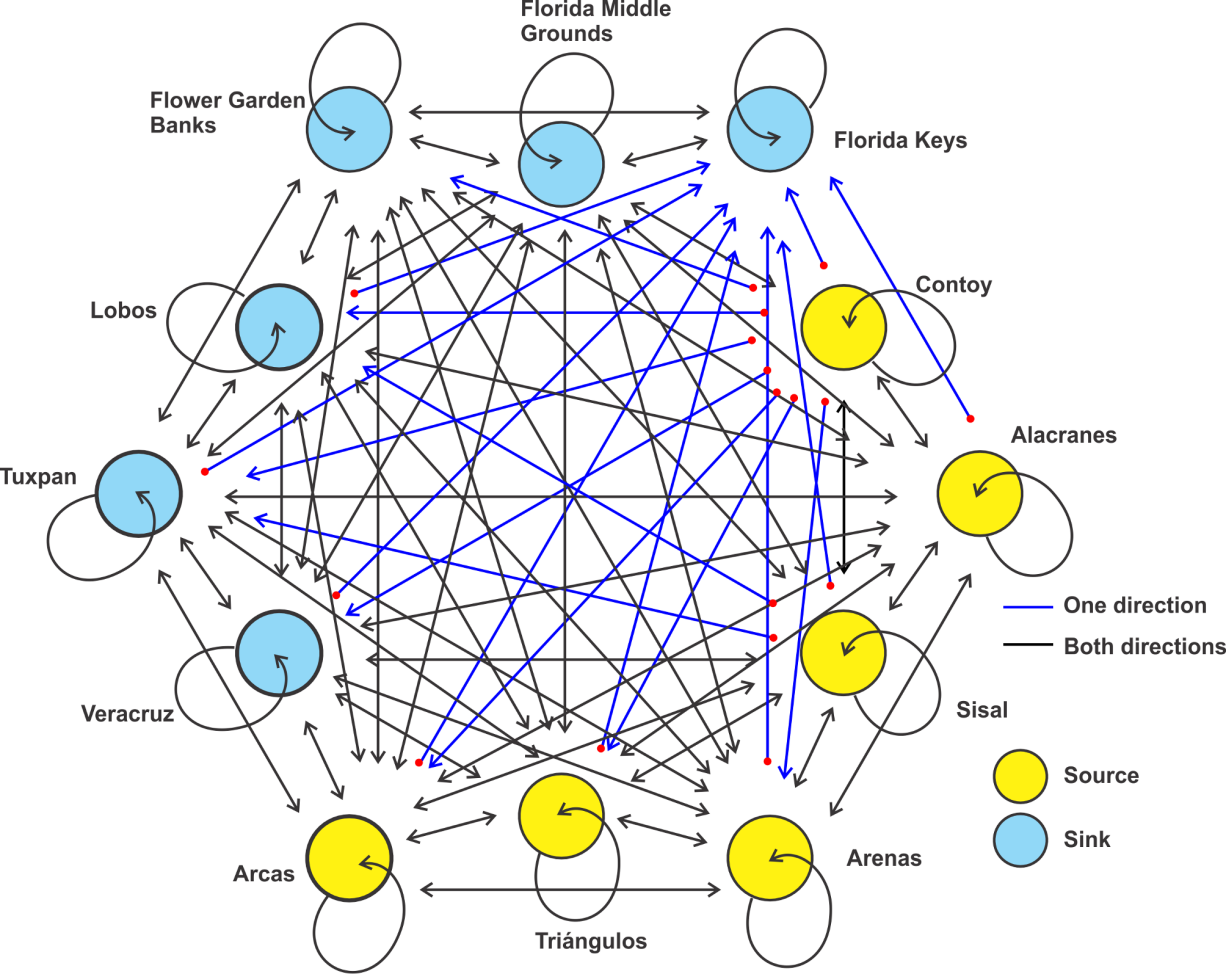
Schematic representation of the metapopulation structure of *Stenopus hispidus* in the Gulf of Mexico.

Most usually, adults are found in crevices at depths of two to four meters and their home-range area is less than a square meter [36]. Data from the GBIF recorded adults on eight reefs of the gulf, from which they should be able to reach the other reefs, and hence hypothetically constitute a large metapopulation in the gulf (Figs 9–11). The metapopulation structure of *S*. *hispidus* seems to fit a patchy model. The source-sink dynamics, in which the number of migrants are higher than those of self-recruits, indicate that connectivity has a fundamental role in the persistence this metapopulation [70]. All six reefs around the Yucatan peninsula function as sources, especially Contoy at the confluence of the Gulf of Mexico and the Caribbean Sea (Fig 11). Thus, *S*. *hispidus* larvae may be transported by the Caribbean Current into the Gulf of Mexico. The large anticyclonic eddies sporadically shed by the Loop Current move westwards and have life spans of months to approximately one year [74]. These eddies are the main means of transport for larvae from the eastern to the western and northern gulf. The time required for drifting particles from Belize to reach Flower Garden Banks is estimated at 55‒135 days [75]; this indicates that taxa with a pelagic larval phase of >2 months may have an important source in the Caribbean Sea.

In the northern gulf, the Flower Garden Banks exhibited its highest connection with Lobos, Tuxpan and Veracruz (Fig 10). In addition to the main current pattern over the shelves already described, tropical storms and hurricanes in the western gulf may induce long-distance dispersal of larvae [76]. For instance, Hurricane Hermine in 2016 induced drifters from the Flower Garden Banks to move about 189 km in ten days owing to coastal currents and southwestward winds [77] because storm winds flow counter-clockwise and induce strong southward winds and currents in the western gulf [76]. Connectivity between the Flower Garden Banks and Florida Middle Banks is limited because of the influence of the Mississippi Canyon and episodic meteorological events on the main current pattern over the shelves [34].

As in the other two species, *S*. *hispidus* showed its highest connectivity and self-recruitment values in Lobos, Tuxpan and Veracruz reefs on the western shelf (Fig 10). Owing to the long dispersal period, the main oceanographic causes of the self-recruitment are presumed to be the seasonal variability in the current direction over the western shelf: southward during the fall‒winter period and northward during spring‒summer [53]. In the northern gulf, the Flower Garden Banks and the Florida Middle Grounds showed the highest self-recruitment (Fig 10). Lugo-Fernández et al. [77], who used observed and simulated drifter trajectories from the Flower Garden Banks, suggested that most drifters (~ 57%) released in the northwestern gulf remain on the shelf, and sometimes drifters may return onto the shelf after several months. On the West Florida Shelf (Florida Middle Grounds), the main cause of higher self-recruitment values could be the changes in current direction: southeastward from October to March (fall‒winter) and northwestward from June to September (summer) [51].

Regarding the different scenarios considered for the competent period of *S*. *hispidus*, results indicate that reefs were also highly connected, but differed in numbers of immigrant larvae. For instance, particles arriving at Tuxpan from the other reefs show that, for a competent window of 44 days, connectivity values are nearly one-half those for the entire period (88 days). For a competent window of ten days, values reduce to about one-fifth (Table 1).

**Table 1:** Percentage of particles successfully arrived at Tuxpan considering the whole competent period of *Stenopus hispidus* and four scenarios.

	First and last days of competent period				
Starting sites	123 to 210	123 to 166	167 to 210	123 to 132	201 to 210
	(88 days)	(44 days)	(44 days)	(10 days)	(10 days)
Florida Keys	0.00	0.00	0.00	0.00	0.00
Florida Middle Grounds	0.06	0.01	0.05	0.00	0.02
Flower Garden Banks	1.45	0.84	0.79	0.30	0.28
Lobos	5.03	2.67	2.87	0.84	1.01
Tuxpan	5.03	2.68	2.85	0.83	1.03
Veracruz	4.24	2.45	2.18	0.81	0.67
Arcas	3.02	1.73	1.60	0.57	0.50
Triángulos	2.74	1.57	1.47	0.51	0.46
Arenas	2.02	1.22	1.03	0.43	0.31
Sisal	2.77	1.62	1.42	0.52	0.46
Alacranes	1.20	0.71	0.61	0.23	0.20
Contoy	1.64	0.80	0.99	0.18	0.29

The earliest recorded settlement time of *S*. *hispidus* is after 123 days in the plankton, but the species is able to delay metamorphosis until suitable environmental conditions and feeding resources are encountered [37, 67]. All the larval dispersal simulations for this species used at least 122 days for the precompetent period, enough time to reach a high degree of connection among reefs. Differences in the competent period were evident in the connectivity values (Table 1). Long-lived dispersive larvae allow populations to reach long distances, increase connections among habitat patches, reduce the probability of inbreeding with the next generation and cope with local extinction [78, 79]. Although long-lived larvae also have high-energy costs related to metamorphosis or its delay in response to specific environmental cues or the lack of them [78], flexibility in the timing of metamorphosis during the competent period may represent a selective advantage because it increases the probability of locating suitable habitats for juveniles and adults [80]. For some invertebrate species, the competent period seems to have profound biogeographical implications as we show below.

### Dispersal scales, pathways and oceanographic barriers

Examination of the physical factors affecting the larval dispersal of each species shows that they operate at different temporal and spatial scales. Physical processes acting in a continuum and juxtaposed scale dimensions largely determine the patterns of plankton distribution [81]. In the Gulf of Mexico, the Loop Current is the most energetic component of the ocean circulation and significantly affects other small-scale hydrodynamic processes [82]. Several studies have demonstrated a high degree of association between the scales at which physical and biological processes occur in the oceans and drive plankton distribution [16, 83, 84]. The findings of this study agree with those previous results. Thus, episodic changes in the current direction over the narrow western shelf derived from the passage of episodic atmospheric systems are presumably the main cause of the high self-recruitment values of *M*. *sculptus*. Instead, the large and long-life eddies shed by the Loop Current seem to be important in the transport of *S*. *hispidus* larvae throughout the Gulf of Mexico.

For the three species, main larval dispersal pathways corresponded to areas over the shelves (Figs 3, 6 and 9). The major concentration of passive particles was over the narrow western shelf, whereas particle distribution was more diluted on the wide shelves bordering the USA. Sanvicente-Añorve et al. [33] argued that over the narrow shelves particles had a high probability of entrapment, especially if there is a decoupling in the current direction between neritic and oceanic zones. Over the shelves, two conspicuous oceanographic features may prevent the transport of larvae: the influence of the deep Mississippi Canyon over the mean current direction in the northern gulf, and the current convergence from October to March in the southernmost region. Gaylord and Gaines [85] indicated that nearshore converging currents may function as oceanographic barriers to larval dispersal and range expansion. At the same time, these barriers may vary in permeability depending on the temporal variability of the nearshore flow and the life-history characteristics of species. In this study, the confluence of currents in the southern gulf is more permeable to *S*. *hispidus*, although *D*. *erythropus* would also be able to cross it. Larval transport through the Mississippi Canyon is limited by the complex bathymetry and by differences in the mean circulation pattern induced by the episodic passage of strong cyclonic mesoscale eddies [34]. In their simulation model, Cardona et al. [34] found that only particles drifting at least one month in the water column can be transported from the Mississippi to the Texas and Louisiana shelves and vice versa. In the present study, only *S*. *hispidus* is able to cross this oceanographic barrier.

### Geographical range versus larval duration

Geographical distribution of the three species here studied seemed to be coupled with their PLD. Whereas the crabs *Mithraculus sculptus* and *Dromia erythropus* are found only in the western Atlantic, the coral shrimp *Stenopus hispidus* has a worldwide distribution. Latitudinally, records of *M*. *sculptus* (25°N to 0°) indicate a distribution slightly more limited than of *D*. *erythropus* (32.3°N to 16°S) [64, 65]. This supports the idea that longer pelagic phases promote wider geographical extents. In spite of the biogeographical and evolutionary importance of this matter, studies of dispersal and distribution in marine invertebrates are relatively few [86, 87]. In a meta-analysis including species of macroalgae, seagrasses, demersal fishes and benthic invertebrates, Lester et al. [31] concluded that PLD is not the primary determinant of the spatial extent of species, but that under some circumstances it can be important. With more restricted and taxonomically comparable datasets, results have been contradictory. Some studies revealed weak positive relationships in Mediterranean littoral fishes [30] and tropical Pacific fishes [27], while others found no relationship [28, 29]. In particular, Wilson and Harrison [88] argued that the long competent period of some coral species influences their widespread distribution in subtropical regions and provides a mechanism whereby they can reach isolated reefs.

It has been suggested that factors that, interacting with the PLD, affect the geographical distribution of species include adult movements, biological traits (fecundity, abundance, niche breadth), ecological behavior, habitat availability, domain of study, center of distribution, and evolutionary ages of species [27, 29, 31, 89–92]. For instance, evolutionary ages may confound the effects of PLD because species with long PLDs may have more restricted geographical occupancy if they are relatively young; in contrast, species with short PLDs may occupy larger areas if they have been dispersing over a long evolutionary time [29]. Statistically, the effect of one variable over a given response can be demonstrated by fixing the remainder explanatory variables. The three species here examined have similar habitat requirements, low motility as adults and pelagic larvae as the main way to disperse [64, 65, 67]; however, many biological and evolutionary aspects remain unknown. Even so, their PLDs seemed to couple with their spatial extent.

## Conclusions

This study examines the influence of hydrodynamic features over the metapopulation structure and larval dispersal pattern of three coral-associated decapod species ‒which depend on their larval phase for dispersal‒ over twelve coral reef systems in the Gulf of Mexico. The case studies analyzed are good examples of how physical and biological processes within the same scale range interact to drive the metapopulation structure of the three species which differ in their PLD. For instance, the synoptic meteorological events (< 15 days) strongly influence the self-recruitment values of *M*. *sculptus* (PLD 8‒13 days), the confluence of opposite currents during the fall‒winter months in the southern gulf impedes connectivity between the southwestern and southeastern reefs in *D*. *erythropus* (28‒30 days), and the long-life eddies (several months) derived from the Loop Current are determinant in the larval transport of *S*. *hispidus* (123‒210 days) from the eastern to the western and northern gulf. Main dispersal pathways of larvae corresponded to neritic waters, especially over the western shelf. In this area, a decoupling in current directions between neritic and oceanic waters during spring-summer is presumably the main cause of particle retention. Two physical features in the gulf may act as oceanographic barriers for larval dispersal: the convergence of currents in the southernmost region during the fall-winter period, and changes in the mean circulation pattern over the Mississippi Canyon due to the complex bathymetry and the influence of aperiodic mesoscale eddies. Only long-lived larvae are able to cross these barriers. The metapopulation structures of *M*. *sculptus* and *D*. *erythropus* seemed to combine characteristics of the patchy and the non-equilibrium models. Instead, the metapopulation structure of *S*. *hispidus* fit better to the patchy model. Analyses of source-sink dynamics of the three species indicated that, while local demographic processes appeared to have the most important role in the persistence of the *M*. *sculptus* metapopulation, dispersal through larvae was the most important for *D*. *erythropus* and *S*. *hispidus* metapopulations. The spatial occupancy of species seems to be coupled with their PLD: a narrower distribution for *M*. *sculptus* and a wider one for *S*. *hispidus*.

## References

[1] Levins R. Extinction. Lectures on mathematics in the life sciences. 1970;2:75–107.

[2] Hanski I, Gilpin M. Metapopulation dynamics: brief history and conceptual domain Metapopulation dynamics: Empirical and theoretical investigations: Elsevier; 1991 p. 3–16.

[3] Hanski I. Metapopulation dynamics. Nature. 1998;396(6706):41.

[4] Jansen VA, Vitalis R. The evolution of dispersal in a Levins’ type metapopulation model. Evolution. 2007;61(10):2386–97. doi: 10.1111/j.1558-5646.2007.00201.x 17714501 10.1111/j.1558-5646.2007.00201.x

[5] Hanski I, Simberloff D. The metapopulation approach, its history, conceptual domain, and application to conservation. Metapopulation biology: Elsevier; 1997 p. 5–26.

[6] Harrison S. Local extinction in a metapopulation context: an empirical evaluation Metapopulation dynamics: empirical and theoretical investigations: Elsevier; 1991 p. 73–88.

[7] Harrison S, Taylor AD. Empirical evidence for metapopulation dynamics Metapopulation biology: Elsevier; 1997 p. 27–42.

[8] Fogarty MJ, Botsford LW. Metapopulation dynamics of coastal decapods. Marine metapopulations. 2006:271–319.

[9] Aiken CM, Navarrete S, Pelegrí J. Potential changes in larval dispersal and alongshore connectivity on the central Chilean coast due to an altered wind climate. Journal of Geophysical Research: Biogeosciences. 2011;116(G4).

[10] Lessios HA, Kessing BD, Robertson DR. Massive gene flow across the world's most potent marine biogeographic barrier. Proceedings of the Royal Society of London B: Biological Sciences. 1998;265(1396):583–8.

[11] Mathews LM. Cryptic biodiversity and phylogeographical patterns in a snapping shrimp species complex. Molecular Ecology. 2006;15(13):4049–63. doi: 10.1111/j.1365-294X.2006.03077.x 17054502 10.1111/j.1365-294X.2006.03077.x

[12] Connolly SR, Baird AH. Estimating dispersal potential for marine larvae: dynamic models applied to scleractinian corals. Ecology. 2010;91(12):3572–83. 21302829 10.1890/10-0143.1

[13] Haye PA, Segovia NI, Muñoz-Herrera NC, Gálvez FE, Martínez A, Meynard A, et al Phylogeographic structure in benthic marine invertebrates of the southeast Pacific coast of Chile with differing dispersal potential. PLoS One. 2014;9(2):e88613 doi: 10.1371/journal.pone.0088613 24586356 10.1371/journal.pone.0088613 PMC3929388

[14] Cowen RK, Sponaugle S. Larval dispersal and marine population connectivity. Annual Review of Marine Science. 2009;1:443–466. doi: 10.1146/annurev.marine.010908.163757 21141044 10.1146/annurev.marine.010908.163757

[15] Cowen RK, Gawarkiewicz G, Pineda J, Thorrold SR, Werner FE. Population connectivity in marine systems an overview. Oceanography. 2007;20(3):14–21.

[16] Sanvicente-Añorve L, Hermoso-Salazar M, Solís-Weiss V, Salgado-Ugarte IH. Carapace relative growth of *Trapezia* Latreille, 1828 (Decapoda, Brachyura), crabs that are symbionts of hard corals, from Clipperton atoll and the Revillagigedo Islands: ecological and zoogeographical implications. Crustaceana. 2010;83(11):1371–83.

[17] Shtilerman E, Stone L, editors. The effects of connectivity on metapopulation persistence: network symmetry and degree correlations. Proc R Soc B; 2015: The Royal Society. 10.1098/rspb.2015.0203 PMC4426627 25833858

[18] Abele LG. Species diversity of decapod crustaceans in marine habitats. Ecology. 1974;55(1):156–61.

[19] Reed JK, Gore RH, Scotto LE, Wilson KA. Community composition, structure, areal and trophic relationships of decapods associated with shallow-and deep-water Oculina varicosa coral reefs: studies on decapod Crustacea from the Indian River region of Florida, XXIV. Bulletin of Marine Science. 1982;32(3):761–86.

[20] Knowlton RE. Larval development of the snapping shrimp *Alpheus heterochaelis* Say, reared in the laboratory. Journal of Natural History. 1973;7(3):273–306.

[21] Booth JD. Jasus edwardsii larval recruitment off the east coast of New Zealand. Crustaceana. 1994;66(3):295–317.

[22] Jackson GA, Strathmann RR. Larval mortality from offshore mixing as a link between precompetent and competent periods of development. The American Naturalist. 1981;118(1):16–26.

[23] Palmas F, Olita A, Addis P, Sorgente R, Sabatini A. Modelling giant red shrimp larval dispersal in the Sardinian seas: density and connectivity scenarios. Fisheries Oceanography. 2017;26(3):364–78.

[24] Pedersen O, Aschan M, Rasmussen T, Tande K, Slagstad D. Larval dispersal and mother populations of *Pandalus borealis* investigated by a Lagrangian particle-tracking model. Fisheries Research. 2003;65(1):173–90.

[25] Butler IV MJ, Paris CB, Goldstein JS, Matsuda H, Cowen RK. Behavior constrains the dispersal of long-lived spiny lobster larvae. Marine Ecology Progress Series. 2011;422:223–37.

[26] Shanks AL. Pelagic larval duration and dispersal distance revisited. The biological bulletin. 2009;216(3):373–85. doi: 10.1086/BBLv216n3p373 19556601 10.1086/BBLv216n3p373

[27] Lester SE, Ruttenberg BI. The relationship between pelagic larval duration and range size in tropical reef fishes: a synthetic analysis. Proceedings of the Royal Society of London B: Biological Sciences. 2005;272(1563):585–91. 10.1098/rspb.2004.2985 PMC1564084 16007745

[28] Zapata FA, Herrón PA. Pelagic larval duration and geographic distribution of tropical eastern Pacific snappers (Pisces: Lutjanidae). Marine Ecology Progress Series. 2002;230:295–300.

[29] Mora C, Treml EA, Roberts J, Crosby K, Roy D, Tittensor DP. High connectivity among habitats precludes the relationship between dispersal and range size in tropical reef fishes. Ecography. 2012;35(1):89–96.

[30] Macpherson E, Raventos N. Relationship between pelagic larval duration and geographic distribution of Mediterranean littoral fishes. Marine Ecology Progress Series. 2006;327:257–65.

[31] Lester SE, Ruttenberg BI, Gaines SD, Kinlan BP. The relationship between dispersal ability and geographic range size. Ecology Letters. 2007;10(8):745–58. doi: 10.1111/j.1461-0248.2007.01070.x 17594430 10.1111/j.1461-0248.2007.01070.x

[32] Johnson DR, Perry HM, Lyczkowski-Shultz J. Connections between Campeche Bank and red snapper populations in the Gulf of Mexico via modeled larval transport. Transactions of the American Fisheries Society. 2013;142(1):50–8.

[33] Sanvicente-Añorve L, Zavala-Hidalgo J, Allende-Arandía M, Hermoso-Salazar M. Connectivity patterns among coral reef systems in the southern Gulf of Mexico. Marine Ecology Progress Series. 2014;498:27–41.

[34] Cardona Y, Ruiz-Ramos DV, Baums IB, Bracco A. Potential connectivity of coldwater black coral communities in the northern Gulf of Mexico. PloS one. 2016;11(5):e0156257 doi: 10.1371/journal.pone.0156257 27218260 10.1371/journal.pone.0156257 PMC4878809

[35] Johnston MW, Bernard AM. A bank divided: quantifying a spatial and temporal connectivity break between the Campeche Bank and the northeastern Gulf of Mexico. Marine Biology. 2017;164(1):12.

[36] Limbaugh C, Pederson H, Chace FA. Shrimps that clean fishes. Bulletin of Marine Science. 1961;11(2):237–57.

[37] Fletcher DJ, Kötter I, Wunsch M, Yasir I. Preliminary observations on the reproductive biology of ornamental cleaner prawns *Stenopus hispidus*, *Lysmata amboinensis* and *Lysmata debelius*. International Zoo Yearbook. 1995;34(1):73–7.

[38] Becker J, Grutter A. Cleaner shrimp do clean. Coral reefs. 2004;23(4):515–20.

[39] Alves DF, Carvalho MC, Barros-Alves SDP, Cobo VJ. Brachyuran crabs (Decapoda, Brachyura) associated with the green sponge *Amphimedon viridis* (Demospongiae) from Itaguá Beach, south-eastern coast of Brazil. Crustaceana. 2012;85(4–5):497–512.

[40] Giese C, Mebs D, Werding B. Resistance and vulnerability of crustaceans to cytolytic sea anemone toxins. Toxicon. 1996;34(8):955–8. 8875782 10.1016/0041-0101(96)00051-7

[41] Calado R, Lin J, Rhyne AL, Araújo R, Narciso L. Marine ornamental decapods—popular, pricey, and poorly studied. Journal of crustacean biology. 2003;23(4):963–73.

[42] Darnell RM, Defenbaugh RE. Gulf of Mexico: Environmental overview and history of environmental research. American Zoologist. 1990:3–6.

[43] Hofmann EE, Worley SJ. An investigation of the circulation of the Gulf of Mexico. Journal of Geophysical Research: Oceans. 1986;91(C12):14221–36.

[44] Duncan CP, Atwood DK, Duncan J, Froelich P. Drift bottle returns from the Caribbean. Bulletin of Marine Science. 1977;27(3):580–6.

[45] Maul GA. The annual cycle of the Gulf Loop Current. Part I: Observations during a one-year time series. J mar Res. 1977;35(1):29–47.

[46] Huh OK, Wiseman WJ, Rouse LJ. Intrusion of Loop Current waters onto the west Florida continental shelf. Journal of Geophysical Research: Oceans. 1981;86(C5):4186–92.

[47] Ohlmann JC, Niiler PP. Circulation over the continental shelf in the northern Gulf of Mexico. Progress in oceanography. 2005;64(1):45–81.

[48] Alvera-Azcárate A, Barth A, Weisberg RH. The surface circulation of the Caribbean Sea and the Gulf of Mexico as inferred from satellite altimetry. Journal of Physical Oceanography. 2009;39(3):640–57.

[49] Morey SL, Martin PJ, O'Brien JJ, Wallcraft AA, Zavala‐Hidalgo J. Export pathways for river discharged fresh water in the northern Gulf of Mexico. Journal of Geophysical Research: Oceans. 2003;108(C10).

[50] Oey LY. Eddy‐and wind‐forced shelf circulation. Journal of Geophysical Research: Oceans. 1995;100(C5):8621–37.

[51] Liu Y, Weisberg RH. Patterns of ocean current variability on the West Florida Shelf using the self‐organizing map. Journal of Geophysical Research: Oceans. 2005;110(C6).

[52] Liddell WD. Origin and geology Coral reefs of the southern Gulf of Mexico Texas A&M University Press, Bryan, TX 2007:23–33.

[53] Zavala‐Hidalgo J, Morey SL, O'Brien JJ. Seasonal circulation on the western shelf of the Gulf of Mexico using a high‐resolution numerical model. Journal of Geophysical Research: Oceans. 2003;108(C12).

[54] Tunnell JW. Coral reefs of the southern Gulf of Mexico: Texas A&M University Press; 2007.

[55] Bleck R. An oceanic general circulation model framed in hybrid isopycnic-Cartesian coordinates. Ocean modelling. 2002;4(1):55–88.

[56] Cummings JA. Operational multivariate ocean data assimilation. Quarterly Journal of the Royal Meteorological Society. 2005;131(613):3583–604.

[57] Marinone S, Gutiérrez O, Parés-Sierra A. Numerical simulation of larval shrimp dispersion in the Northern Region of the Gulf of California. Estuarine, Coastal and Shelf Science. 2004;60(4):611–7.

[58] Döös K, Engqvist A. Assessment of water exchange between a discharge region and the open sea–a comparison of different methodological concepts. Estuarine, Coastal and Shelf Science. 2007;74(4):709–21.

[59] Döös K, Rupolo V, Brodeau L. Dispersion of surface drifters and model-simulated trajectories. Ocean Modelling. 2011;39(3):301–10.

[60] Metzger E, Hurlburt H, Wallcraft A, Shriver J, Smedstad L, Smedstad O, et al. Validation Test Report for the Global Ocean Prediction System V3. 0-1/12 Degree HYCOM/NCODA: Phase I. Naval Research Lab Stennis Space Center Ms Oceanography Div, 2008.

[61] Zelenke B, Moline MA, Crawford GB, Garfield N, Jones BH, Largier JL, et al., editors. Evaluating connectivity between marine protected areas using CODAR high-frequency radar. OCEANS 2009, MTS/IEEE Biloxi-Marine Technology for Our Future: Global and Local Challenges; 2009: IEEE.

[62] Sanvicente-Añorve L, Alatorre M, Flores-Coto C, Alba C. Relationships between fish larvae and siphonophores in the water column: effect of wind-induced turbulence and thermocline depth. ICES Journal of Marine Science. 2007;64(5):878–88.

[63] Xue H, Incze L, Xu D, Wolff N, Pettigrew N. Connectivity of lobster populations in the coastal Gulf of Maine: Part I: Circulation and larval transport potential. ecological modelling. 2008;210(1):193–211.

[64] Colin PL. Marine invertebrates and plants of the living reef. TFH Publications 1988.

[65] Manning RB, Chace FA. Decapod and stomatopod crustacea from Ascension Island, South Atlantic Ocean. Smithsonian contributions to zoology (USA). 1990.

[66] Viana G, Ramos-Porto M, Travassos P, Carvalho G. Registro de Dromia erythropus (G. Edwards, 1771) para o Arquipélago de Fernando de Noronha, Brasil (Crustacea, Decapoda, Dromiidae). Bol téc cient CEPNOR, Belém. 2003;3(1):215–8.

[67] Chockley BR, Mary CMS. Effects of body size on growth, survivorship, and reproduction in the banded coral shrimp, *Stenopus hispidus*. Journal of crustacean biology. 2003;23(4):836–48.

[68] Laughlin RA, Rodríguez PJ, Marval JA. The complete larval development of the sponge crab *Dromia erythropus* (George Edwards, 1771)(Brachyura: Dromiidae) from the Archipielago de los Roques, Venezuela. Journal of Crustacean Biology. 1982;2(3):342–59.

[69] Rhyne AL, Fujita Y, Calado R. Larval development and first crab of *Mithraculus sculptus* (Decapoda: Brachyura: Majoidea: Mithracidae) described from laboratory-reared material. Journal of the Marine Biological Association of the United Kingdom. 2006;86(5):1133–47.

[70] Puckett B, Eggleston D. Metapopulation dynamics guide marine reserve design: importance of connectivity, demographics, and stock enhancement. Ecosphere. 2016;7(6). doi: 10.1002/ecs2.1368

[71] Allende-Arandía ME, Zavala-Hidalgo J, Romero-Centeno R, Mateos-Jasso A, Vargas-Hernández JM, Zamudio L. Analysis of ocean current observations in the northern Veracruz Coral Reef System, Mexico: 2007–12. Journal of Coastal Research. 2015;32(1):46–55.

[72] Gd Melo, Campos O Jr. A família Dromiidae De Haan no litoral brasileiro, com descrição de uma nova espécie (Crustacea: Decapoda: Brachyura). Revista Brasileira de Zoologia. 1999;16(2):273–91.

[73] Roberts CM. Connectivity and management of Caribbean coral reefs. Science. 1997;278(5342):1454–7. 9367956 10.1126/science.278.5342.1454

[74] Oey LY, Ezer T, Lee HC. Loop Current, rings and related circulation in the Gulf of Mexico: A review of numerical models and future challenges. Circulation in the Gulf of Mexico: Observations and models. 2005:31–56.

[75] Lugo-Fernández A. Travel times of passive drifters from the Western Caribbean to the Gulf of Mexico and Florida-Bahamas. Gulf of Mexico Science. 2006;24(1/2):61.

[76] Lugo-Fernandez A, Gravois M. Understanding impacts of tropical storms and hurricanes on submerged bank reefs and coral communities in the northwestern Gulf of Mexico. Continental Shelf Research. 2010;30(10):1226–40.

[77] Lugo-Fernández A, Deslarzes KJ, Price JM, Boland GS, Morin MV. Inferring probable dispersal of Flower Garden Banks coral larvae (Gulf of Mexico) using observed and simulated drifter trajectories. Continental Shelf Research. 2001;21(1):47–67.

[78] Pechenik JA. On the advantages and disadvantages of larval stages in benthic marine invertebrate life cycles. Marine Ecology Progress Series. 1999:269–97.

[79] Treml EA, Ford JR, Black KP, Swearer SE. Identifying the key biophysical drivers, connectivity outcomes, and metapopulation consequences of larval dispersal in the sea. Movement ecology. 2015;3(1):17 doi: 10.1186/s40462-015-0045-6 26180636 10.1186/s40462-015-0045-6 PMC4502943

[80] Gebauer P, Paschke K, Anger K. Delayed metamorphosis in decapod crustaceans: evidence and consequences. Revista Chilena de Historia Natural. 2003;76:169–75.

[81] Haury L, McGowan J, Wiebe P. Patterns and processes in the time-space scales of plankton distributions Spatial pattern in plankton communities: Springer; 1978 p. 277–327.

[82] Lin L, Zhuang W, Huang Y. Lagrangian Statistics and Intermittency in Gulf of Mexico. Scientific reports. 2017;7(1):17463 doi: 10.1038/s41598-017-17513-9 29234051 10.1038/s41598-017-17513-9 PMC5727302

[83] Sanvicente-Añorve L, Flores-Coto C, Chiappa-Carrara X. Temporal and spatial scales of ichthyoplankton distribution in the southern Gulf of Mexico. Estuarine, Coastal and Shelf Science. 2000;51(4):463–75.

[84] Cowen RK, Paris CB, Srinivasan A. Scaling of connectivity in marine populations. Science. 2006;311(5760):522–7. doi: 10.1126/science.1122039 16357224 10.1126/science.1122039

[85] Gaylord B, Gaines SD. Temperature or transport? Range limits in marine species mediated solely by flow. The American Naturalist. 2000;155(6):769–89. doi: 10.1086/303357 10805643 10.1086/303357

[86] Perron F, Kohn A, editors. Larval dispersal and geographic distribution in coral reef gastropods of the genus Conus. Proc Fifth Int Coral Reef Congr, Tahiti; 1985.

[87] Yednock BK, Neigel JE. Rethinking the mechanisms that shape marine decapod population structure. Phylogeography and Population Genetics in Crustacea. 2011:57–73.

[88] Wilson J, Harrison PL. Settlement-competency periods of larvae of three species of scleractinian corals. Marine Biology. 1998;131(2):339–45.

[89] Treml EA, Roberts JJ, Chao Y, Halpin PN, Possingham HP, Riginos C. Reproductive output and duration of the pelagic larval stage determine seascape-wide connectivity of marine populations. Oxford University Press; 2012. 10.1093/icb/ics101 22821585

[90] Luiz OJ, Allen AP, Robertson DR, Floeter SR, Kulbicki M, Vigliola L, et al Adult and larval traits as determinants of geographic range size among tropical reef fishes. Proceedings of the National Academy of Sciences. 2013;110(41):16498–502. 10.1073/pnas.1304074110 PMC3799316 24065830

[91] Kulbicki M. Increase in pelagic larval duration and geographical range for reef fish along an oceanic gradient. Vie et Milieu = Life and Environment. 2015;65(1):1–9.

[92] Gaither MR, Bowen BW, Rocha LA, Briggs JC. Fishes that rule the world: circumtropical distributions revisited. Fish and Fisheries. 2016;17(3):664–79.

